# Study on the Effects of Vibration Force Field on the Mixing and Structural Properties of PLA/PBS/EGMA Blends

**DOI:** 10.3390/polym17070947

**Published:** 2025-03-31

**Authors:** Bin Xue, Jun Li, Qu Yang, Danxiang Wei, Guiting Wu

**Affiliations:** 1 School of Mechanical and Automotive Engineering, Guangxi University of Science and Technology, Liuzhou 545616, China; 19966407796@163.com (J.L.); weidanxiang@gmail.com (D.W.); wuguiting0122@163.com (G.W.); 2 School of Mechanical and Marine Engineering, Beibu Gulf University, Qinzhou 535011, China

**Keywords:** PLA, PBS, EGMA, vibration force field, polymer blends, mechanical properties, thermal stability, crystallization

## Abstract

This study investigates the effects of a vibration force field on the mixing and structural properties of polylactic acid (PLA), polybutylene succinate (PBS), and ethylene–glycidyl methacrylate terpolymer (EGMA) blends. A balanced triple-screw dynamic extrusion process was utilized to prepare PLA/PBS/EGMA composites under various vibration parameters, specifically amplitude and frequency. The results indicate that the introduction of a vibration force field significantly enhances the dispersion of the PLA/PBS/EGMA blend, leading to improved mechanical properties, thermal stability, and crystallization behavior. When the vibration frequency was 6 Hz and the amplitude was 1.0 mm, the impact strength increased from the steady-state value of 70.86 KJ/m^2^ to 88.21 KJ/m^2^. When the amplitude was 0.4 mm and the frequency was 10 Hz, the impact strength reached 81.86 KJ/m^2^. The orthogonal experimental design and entropy method analysis revealed that vibration frequency and amplitude play a dominant role in optimizing mechanical performance, whereas processing temperature and rotor speed exhibit minimal impact. Scanning electron microscopy (SEM) analysis confirmed that the vibration force field reduces phase separation, promoting a finer and more homogeneous dispersion of PBS and EGMA within the PLA matrix. Additionally, TGA and DTG curves suggest that when the vibration amplitude and frequency are lower than specific thresholds, the thermal stability of the blend deteriorates. In contrast, when they exceed those thresholds, thermal stability improves. For instance, with an amplitude of 1.0 mm, the initial degradation temperature (T5) climbs from 328.6 °C to 333.7 °C. At a frequency of 10 Hz, T5 reaches 333.1 °C. These findings provide theoretical support for the application of vibration-assisted extrusion in the development of high-performance biodegradable polymer blends.

## 1. Introduction

In recent years, the growing severity of global environmental issues has led to increasing interest in the development and application of biodegradable polymers [1,2,3,4,5]. Among them, polylactic acid (PLA) and polybutylene succinate (PBS) have emerged as two highly promising biodegradable materials [6,7,8,9,10,11]. PLA, derived from renewable resources such as corn starch, sugarcane, and cassava, exhibits high tensile strength, excellent rigidity, and outstanding processability [12,13,14]. However, its brittleness and slow crystallization rate limit its broader application [15,16,17]. In contrast, PBS offers superior flexibility, high elongation at break, and excellent biodegradability under various environmental conditions [18,19,20,21]. By blending PLA with PBS, it is possible to develop a composite material that combines the advantages of both polymers, thereby enhancing mechanical properties and biodegradability [22,23,24,25,26].

In polymer blend systems, the addition of a compatibilizer is often essential for improving the compatibility between different polymer phases [27,28,29,30,31]. Ethylene–glycidyl methacrylate terpolymer (EGMA) is widely used as a compatibilizer in polymer blends [32,33]. Compared with the mixture of maleic anhydride and styrene-based compatibilizers, (EGMA) shows better results, and the mechanical properties of the copolymer are superior. Its epoxy groups can react with the terminal groups of PLA and PBS, forming covalent bonds that enhance interfacial adhesion and reduce phase separation, thereby improving the overall properties of the blend [23,34]. During the processing of polymer blends, the mixing and dispersion of different phases play a critical role in determining the final properties of the composite material. In traditional screw extruders, material mixing mainly relies on the shearing action generated by the rotation of screws. The shear flow field during this process plays a dominant role. When a vibration force field is applied to the screws, a tensile flow field is generated. Thus, the materials are mixed under the influence of both shear and tensile flow fields, leading to improved mixing results. Therefore, introducing a vibration force field during polymer processing has emerged as a promising strategy [35,36]. This approach not only enhances the mixing and dispersion efficiency of the polymer melt but also influences the morphology, crystallization behavior, and mechanical properties of the final product [37,38,39]. By adjusting vibration parameters such as amplitude and frequency, the mixing process and material performance can be more precisely controlled [40,41].

In this study, a novel extrusion process was used to prepare a three-phase blend of polylactic acid (PLA), polybutylene succinate (PBS), and ethylene–glycidyl methacrylate terpolymer (EGMA). This novel preparation method, by precisely adjusting vibration parameters like amplitude and frequency, is capable of increasing the stretching flow field during the original mixing process. Consequently, it not only improves the mixing and dispersion efficiency of the polymer melt, but also exerts a substantial influence on the morphology, crystallization behavior, and mechanical properties of the final product. The effects of vibration force field parameters, specifically amplitude and frequency, on the mixing and blending performance were investigated. By introducing a vibration force field, we examined its impact on the dispersion of the PLA/PBS/EGMA blend and analyzed how dispersion efficiency influences the mechanical properties, microstructure, thermal stability, and crystallization behavior of the material. The findings provide theoretical support and guidance for the future development of multiphase polymer blends using a balanced triple-screw dynamic extrusion system.

## 2. Materials and Methods

### 2.1. Materials

Polylactic acid (PLA): Grade 4032D, with a density of 1.25 g/cm^3^ and a D-isomer lactide content of 1.2–1.6 wt%, supplied by Nature Works LLC (Minneapolis, MI, USA).

Polybutylene succinate (PBS): Grade 3001MD, with a melting temperature of 110–130 °C, crystallinity of 30–45%, and a density of 1.26 g/cm^3^, supplied by Showa High Polymer Co., Ltd. (Tokyo, Japan).

Ethylene–glycidyl methacrylate-methyl acrylate terpolymer (EGMA): Grade LOTADER AX 8900, with a density of 0.944 g/cm^3^, containing 24 wt% methyl acrylate and 8 wt% glycidyl methacrylate, supplied by Arkema Investment Co., Ltd. (Beijing, China).

### 2.2. Experimental Plan

Pre-drying process: PLA was dried in an oven at 80 °C for 10 h, while PBS and EGMA granules were dried separately at 50 °C for 10 h.

Preparation of PLA/PBS/EGMA blends: The blends were prepared using a balanced triple-screw dynamic extruder, with component mass fractions of 100/0/0, 90/10/0, 86/10/4, 82/10/8, and 78/10/12. The extruder temperatures were set at 170-180-190-190-190-190-190-190-180-180 °C from the hopper to the die. The rotor speed was 80 rpm, and the feeder screw speed was 6 rpm. The extruded blends were then dried in an oven at 80 °C for 10 h before being molded into standard dumbbell-shaped and rectangular specimens using a vulcanizing press at 195 °C and 15 MPa for tensile and impact testing.

Optimization and statistical analysis: Based on the mechanical performance results, the optimal blend composition was selected. Orthogonal experiments were conducted to analyze the effects of vibration field parameters and processing conditions on the mechanical properties of the blends. The impact of these parameters on tensile strength, elongation at break, and impact strength was investigated. The entropy method was used to determine the weight of each performance indicator, followed by a multi-criteria comprehensive evaluation to calculate the overall score. Variance analysis and range analysis were also performed.

Single-factor analysis: Based on the results of the orthogonal experiments, a single-factor analysis was conducted on the most significant influencing parameters to further investigate the effect of processing conditions on the PLA/PBS/EGMA blend system.

### 2.3. Experiment

#### 2.3.1. Equipment and Instruments

Balanced triple-screw dynamic extruder (Model: R-MOM-75), independently developed by South China University of Technology. The important instrument used for mixing materials in this thesis.

Plate vulcanizing machine (Model: QLB-25D/Q), manufactured by Wuxi First Rubber & Plastic Machinery Co., Ltd. (Wuxi, China). It is mainly used for the vulcanization and molding of materials such as rubber and plastics. By applying a certain amount of pressure and temperature to the materials inside the mold and maintaining them for a specific period of time, a crosslinking reaction occurs in the materials. As a result, vulcanized products with specific shapes, dimensions, and properties are obtained.

Scanning electron microscope (SEM) (Model: Quanta FEG250), supplied by FEI Company (Hillsboro, OR, USA). By using a focused electron beam to scan the surface of the sample, signals such as secondary electrons and backscattered electrons are generated. This enables the acquisition of high-resolution images of the sample’s surface. These images can be used to observe the microscopic morphology, structural characteristics, particle size, and particle distribution of the materials.

Fourier transform infrared spectrometer (FT-IR) (Model: Nicolet Nexus 670), provided by Thermo Nicolet Corporation (Waltham, MA, USA). By measuring the absorption degree of the sample to infrared light of different wavelengths, the infrared absorption spectrum of the sample can be obtained. Since different functional groups have specific absorption peaks in the infrared spectrum, this method can be used to analyze the chemical structure of the sample, the types of chemical bonds, and so on.

Differential scanning calorimeter (DSC) (Model: DSC 204), manufactured by NETZSCH (Selb, Germany). Under the condition of programmed temperature control, the relationship between the power difference input into the sample and the reference material and the change of temperature or time is measured. It can be used to study the phase change, melting, crystallization, thermal stability, chemical reaction heat, etc., of materials.

Thermogravimetric analyzer (TGA) (Model: TG 209F3), supplied by NETZSCH (Selb, Germany). In a programmed temperature-rising environment, the change in the mass of the sample with temperature or time is measured. It is mainly used to study the thermal stability of materials, the thermal decomposition process, the oxidative degradation behavior, and the content of volatile components in materials.

Pendulum impact tester (Model: Zwick 5117), manufactured by Zwick GmbH (Ulm, Germany). A pendulum is allowed to swing down freely to impact the sample. As a result, the sample breaks instantaneously under the impact load. By measuring the change in the pendulum’s energy before and after the impact, the impact strength of the material can be calculated. This calculation helps to evaluate the material’s ability to resist impact damage. This instrument is one of the important devices for testing the mechanical properties of materials.

#### 2.3.2. Sample Preparation and Characterization

Fourier Transform Infrared (FT-IR) spectroscopy (Nicolet Nexus 670, Thermo Fisher Scientific Inc, Waltham, MA, USA) was used to investigate potential compatibility reactions between the materials. Samples were prepared into thin films using a hot-pressing method. FT-IR spectra were recorded for all tested samples using the attenuated total reflectance (ATR) mode. The spectra were obtained with 64 scans over a wavenumber range of 400 cm⁻^1^ to 4000 cm⁻^1^, with a resolution of 8 cm⁻^1^.

The microstructure of the composite blend system was examined using scanning electron microscopy (SEM) (Quanta FEG250, FEI company, Hillsboro, OR, USA). The prepared samples were immersed in liquid nitrogen for 30 min, then fractured in a brittle manner and coated with gold. SEM imaging was conducted at an accelerating voltage of 5 kV and a working distance of 11 mm. Prior to observation, all samples underwent an etching process. The etching conditions were as follows: samples were immersed in an etching solution (40 mg sodium hydroxide, 13 mL methanol, and 27 mL distilled water) at 25 °C for 12 h. They were then rinsed thoroughly with water for five minutes and air-dried at room temperature for six hours.

The tensile and impact properties of the samples were measured using an Instron universal testing machine (Model 5566, INSTRON company, Norwood, MA, USA) and a PIT501B-Z pendulum impact tester (Zwick 5117, Zwick GmbH, Ulm, Germany), respectively. The tensile test was conducted according to the GB/T 1040-2006 standard, with a crosshead speed of 20 mm/min (equivalent to 100% strain per minute). Impact strength was measured following the GB/T 1843-2008 standard. The mechanical test results for each group were averaged over five repeated measurements.

The crystallization behavior of the blend/composite system was analyzed using differential scanning calorimetry (DSC) (Netzsch Group, Selb, Germany) under a nitrogen atmosphere. To eliminate thermal history effects, samples weighing 5–10 mg were first heated from 30 °C to 200 °C at a rate of 10 °C/min and held at 200 °C for 4 min. They were then cooled back to 30 °C at the same rate, followed by a second heating cycle at 10 °C/min up to 200 °C. The second heating scan was used to determine the melting temperature (Tm), cold crystallization temperature (Tcc), and enthalpy of fusion (Δ*Hm*). The degree of crystallinity (*λc*) was calculated using Equation (1):

(1) $$\lambda_{c}=\frac{\Delta H_{m}-\Delta H_{cc}}{\Delta H_{c}^{t}\cdot \omega }$$

In the equation, *λ_c_* represents the crystallinity of PLA, ω is the mass fraction of PLA, and Δ$H_{c}^{t}$ is the theoretical enthalpy of fusion for fully crystalline LDPE (Δ$H_{c}^{t}$ = 93.6 J/g).

The thermal stability of the blend system was analyzed using a Netzsch TG209 thermogravimetric analyzer under a nitrogen atmosphere. The samples were heated from 30 °C to 600 °C at a rate of 10 °C/min to prevent oxidation. Approximately 6–10 mg of each sample was used for testing.

The degree of reaction in the ternary blend was evaluated by measuring the gel fraction (Gf). Approximately 1 g of the impact-tested sample was cut and dissolved in chloroform at room temperature for 24 h. The resulting solution was separated using high-speed centrifugation, followed by filtration through filter paper to collect the gel. The gel fraction (Gf) was calculated using Equation (2).

(2) $$\mathrm{Gel}\;\mathrm{fraction}\left(\%\right)=\frac{W_{2}-W_{1}}{W_{0}}\times 100\%$$

In the equation, *W*_0_ represents the initial weight of the sample, *W*_1_ is the weight of the filter paper, and *W*_2_ is the total weight of the extracted gel and filter paper after drying in a vacuum oven at 80 °C for 6 h.

## 3. Results and Discussion

### 3.1. Research on the Influence of Vibration Force Field on Mechanical Properties

This section investigates the effects of vibration force field parameters and processing conditions on the PLA/PBS/EGMA (82/10/8) blend system. Using an orthogonal experimental design, the study evaluates the impact of these parameters on mechanical properties, specifically tensile strength, elongation at break, and impact strength. The entropy method is applied to determine the weight of each evaluation index, while a multi-criteria comprehensive evaluation method is used to calculate the overall score of the indicators. Variance analysis and range analysis are then conducted to assess the influence of each parameter comprehensively. In the experiment, processing temperature, screw speed, vibration frequency, and vibration amplitude are defined as influencing factors A, B, C, and D, respectively, with each factor set at four levels, as shown in Table 1. Assuming no interaction between these factors, an orthogonal test table L16(4^5^) is designed (Table 2) to systematically evaluate the impact of processing conditions on the mechanical properties of the blend system.

The comprehensive score of a single test = the weight of tensile strength membership × tensile strength + the weight of elongation at break × elongation at break + the weight of impact strength membership × impact strength.

The tensile strength weights are 0.10, the weights of elongation at break are 0.07, and the weights of impact strength are 0.83, respectively, and the orthogonal test results and comprehensive scoring results are shown in Table 2.

By calculating the range of each column in the range analysis, a larger range value indicates a greater influence on the experimental results. This allows us to determine the relative importance of each factor in affecting the test outcomes. The range analysis results for each influencing factor are shown in Table 2. The results indicate that Rc > Rd > Rb > Ra, meaning the order of influence on the overall mechanical performance is vibration frequency, vibration amplitude, rotation speed, and temperature.

The range analysis identifies the relative importance of each influencing factor on the overall mechanical performance of the blend. However, it does not quantify the extent of their impact. Therefore, to assess the degree to which vibration frequency, vibration amplitude, rotation speed, and temperature affect the evaluation metrics, variance analysis is conducted in this section. The $F_{\alpha }$ follows an F-distribution with degrees of freedom ($df_{\alpha }$, $df_{E}$). For a given significance level of α = 0.05, if $F_{\alpha }$ > ($df_{\alpha }$, $df_{E}$), it indicates that the factor has a significant impact on the experimental results. As shown in Table 3, the processing parameters $F_{A,B}$ < $F_{\alpha }$ ($df_{\alpha }$, $df_{E}$) = 9.280, $F_{C,D}$ < $F_{\alpha }$ ($df_{\alpha }$, $df_{E}$) = 9.280, indicate that processing temperature and rotation speed have no significant impact on the overall mechanical properties of the PLA/PBS/EGMA blend system, whereas vibration amplitude and frequency have a significant effect.

### 3.2. Research on the Impact of Vibration Force Field on System Performance

The variance analysis of the orthogonal experiment indicates that both amplitude and frequency have a significant impact on the overall mechanical properties of the PLA/PBS/EGMA blend system. To further investigate the effects of vibration on the dispersion and other properties of the system, frequency and amplitude were separately varied to prepare the blend system, and the impact of the vibration force field on the performance of the blend was analyzed. Since the processing temperature and screw speed do not significantly influence the comprehensive mechanical properties of the PLA/PBS/EGMA blend system, these two parameters are fixed in this section. Based on the range analysis results from Table 2, optimal mechanical properties are achieved under processing conditions of 200 °C for temperature and 80 rad/s for screw speed. Therefore, these conditions were selected and fixed for the experimental study in this section.

#### 3.2.1. The Influence of Vibration Force Field on the Micro-Morphology of the System

It is well known that the phase morphology, particle size, and distribution of the dispersed phase in a multiphase blend system significantly affect the performance of the final product. To further investigate the relationship between the vibration force field generated by the self-developed balanced triple-screw dynamic extruder and the mechanical performance and microstructural evolution of the PLA/PBS/EGMA ternary blend, scanning electron microscopy (SEM) was used to observe the fracture surface morphology under different vibration frequencies and amplitudes, specifically focusing on both brittle and impact fracture surfaces.

**Figure 1:**
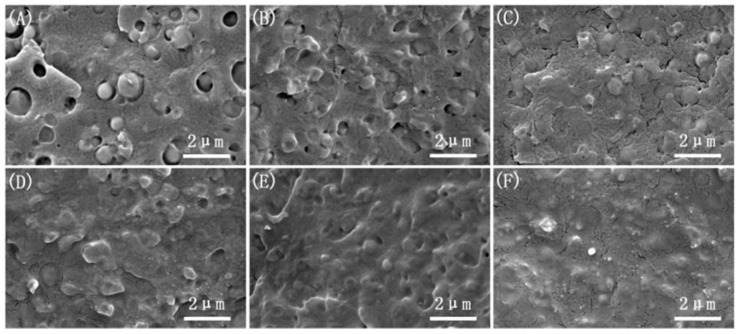
Microscopic morphology of the cryofracture surfaces with different vibration amplitudes: (**A**) F0H0; (**B**) F6H0.2; (**C**) F6H0.4; (**D**) F6H0.6; (**E**) F6H0.8; (**F**) F6H1.0.

The microstructure of the brittle fracture surface under different amplitudes is shown in Figure 1. Under steady-state operating conditions, as shown in Figure 1A, the brittle fracture surface exhibits a distinct “sea-island” structure, with PBS dispersed as spherical particles within the PLA matrix. The interface between the particles and the matrix is clearly visible, and there is a significant gap between the two phases. The particle size of PBS is relatively large and unevenly distributed, resulting in poor dispersion without the introduction of the vibration force field. This is similar to the electron microscopy results obtained by Ullah [23] in the previous text.

When the vibration force field is applied (with a fixed frequency of F = 6 Hz and an amplitude of H = 0.2 mm), as shown in Figure 1B, most of the interface gaps between the PBS particles and the PLA matrix disappear. The particle size of the spherical PBS decreases, and the distribution becomes more uniform. The microstructure of the brittle fracture surface shows significant improvement. As the amplitude continues to increase, as shown in Figure 1C–F, the fracture surface becomes progressively smoother. The “sea-island” structure gradually disappears, and the PBS particle size decreases further, with an increasingly uniform distribution. Particularly, when the amplitude is H = 1.0 mm, the brittle fracture surface of the ternary system exhibits a co-continuous phase morphology, indicating that the introduction of amplitude enhances the compatibility of the system. This is primarily due to the cyclic shear-stretching composite flow field created by the amplitude, which exerts strong shear, stretching, and compression forces on the particles in the extruder barrel. These forces result in smaller and more uniform particles, increasing the probability of particle contact, promoting in situ reactions between particles, improving compatibility, and enhancing phase morphology.

**Figure 2:**
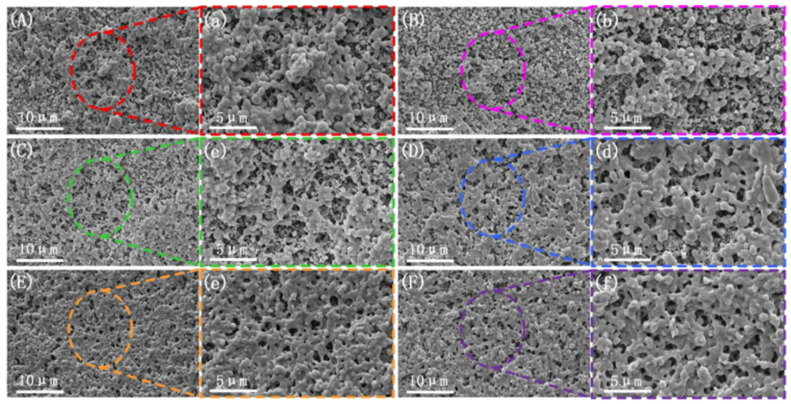
Microscopic morphology of the cryofracture surfaces PLA amorphous were etched with different vibration amplitudes: (**A**,**a**) F0H0; (**B**,**b**) F6H0.2; (**C**,**c**) F6H0.4; (**D**,**d**) F6H0.6; (**E**,**e**) F6H0.8; (**F**,**f**) F6H1.0.

The scanning electron microscope (SEM) images of the brittle fracture surface above show that the microstructure improves significantly under the application of amplitude. However, the specific relationship between the phases under the influence of amplitude remains unclear. To further investigate the effect of amplitude on the phase morphology, a sodium hydroxide–methanol–water solution was used to etch the surface of the frozen brittle fracture, removing the non-crystalline PLA phase in the blend [40]. The microstructure of the PLA/PBS/EGMA blend under steady-state and varying amplitude conditions is shown in Figure 2. From Figure 2A,a, it can be observed that under steady-state conditions, large PBS and EGMA particles are clearly visible on the etched brittle fracture surface. EGMA connects the PBS and non-crystalline PLA phases, acting as a bridge. However, there is a significant amount of particle aggregation, indicating poor dispersion under steady-state conditions. When the vibration force field is applied (with a fixed vibration frequency of F = 6 Hz), as the amplitude increases, the phase morphology on the etched brittle fracture surface shows a clear improvement. The aggregation of particles gradually decreases and eventually disappears. Particularly, when the amplitude exceeds 1.0 mm, the particle size decreases, and the three-phase materials form a network-like structure, indicating that the introduction of amplitude enhances dispersion. This enables full contact between the three phases, promoting in situ reactions and improving interfacial compatibility.

**Figure 3:**
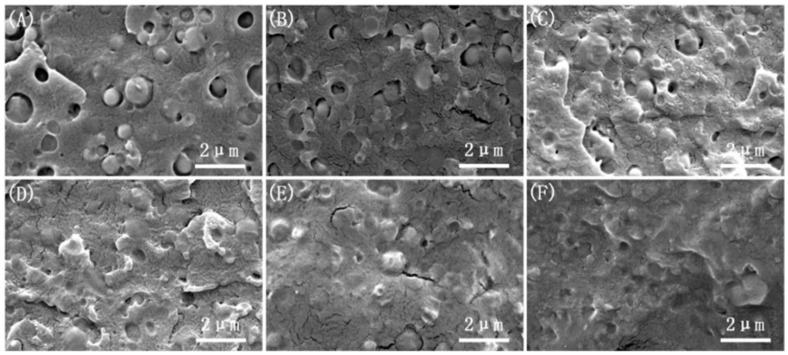
Microscopic morphology of the cryofracture surfaces with different vibration frequencies: (**A**) F0H0; (**B**) H0.4F2; (**C**) H0.4F4; (**D**) H0.4F6; (**E**) H0.4F8; (**F**) H0.4F10.

The microstructure of the brittle fracture surface under different vibration frequencies is shown in Figure 3. When the vibration force field is applied (with a fixed amplitude of H = 0.4 mm), as the frequency increases, the “sea-island” structure on the microstructure gradually disappears. The size of the dispersed PBS spherical particles decreases, and their distribution becomes more uniform, leading to a clear improvement in compatibility. Particularly, when the frequency is F = 10 Hz, as shown in Figure 2F, the microstructure of the brittle fracture surface becomes smoother, suggesting that the increase in vibration frequency enhances the compatibility of the PLA/PBS/EGMA blend. Compared with the effect of amplitude on the brittle fracture surface microstructure in Figure 1, it is evident that vibration frequency and amplitude have similar effects on dispersion. The main reason is that as the frequency increases, the number of “stretching–compression” cycles per unit time increases. Additionally, the shear flow field generated by the rotation of the three-screw extruder enhances the mixing and kneading ability of the polymer equipment, improving the overall mixing efficiency.

**Figure 4:**
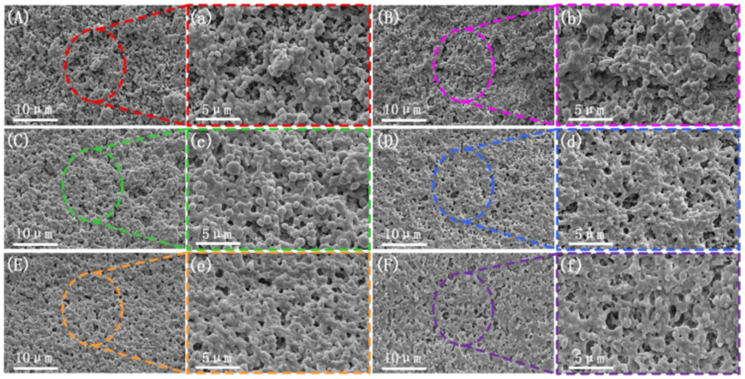
Microscopic morphology of the cryofracture surfaces with different vibration frequencies: (**A**,**a**) F0H0; (**B**,**b**) H0.4F2; (**C**,**c**) H0.4F4; (**D**,**d**) H0.4F6; (**E**,**e**) H0.4F8; (**F**,**f**) H0.4F10.

To further investigate the impact of vibration frequency on the phase morphology of the PLA/PBS/EGMA blend, the brittle fracture surfaces at different vibration frequencies were also subjected to etching treatment. The microstructure is shown in Figure 4.

From the observation of the microstructure, when the vibration force field is applied (with a fixed amplitude of H = 0.4 mm), as the frequency increases, a noticeable improvement in the phase morphology is observed when compared to the microstructure of the blend prepared under steady-state conditions. This is similar to Qu’s research [39]. Large-scale aggregation of PBS and EGMA particles gradually disappears with the increase in vibration frequency. Additionally, the dispersed phase becomes more uniformly distributed within the PLA matrix, confirming that an increase in vibration frequency can also improve the mixing and kneading effect.

**Figure 5:**
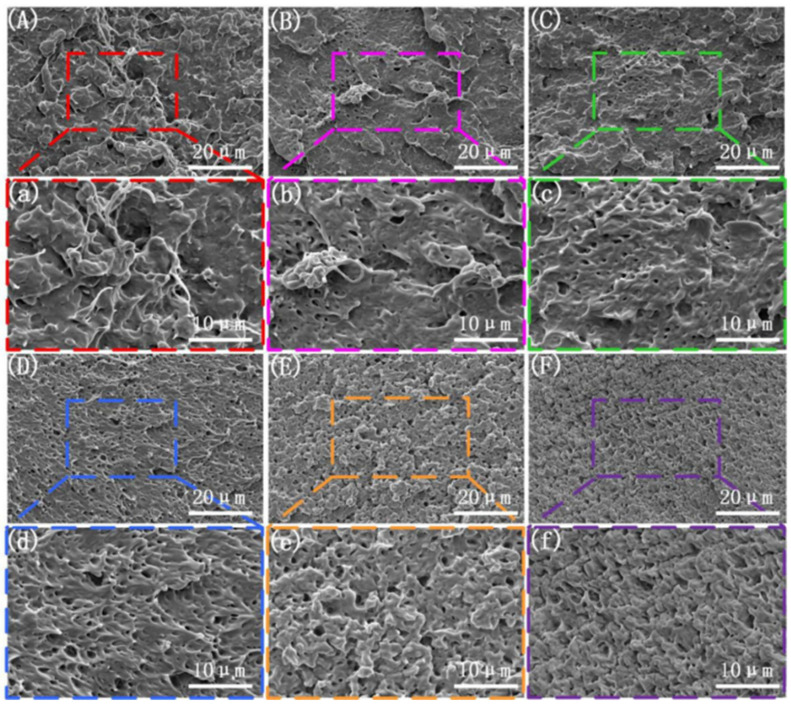
Microscopic morphology of impact fracture surfaces with different vibration amplitudes: (**A**,**a**) F0H0; (**B**,**b**) F6H0.2; (**C**,**c**) F6H0.4; (**D**,**d**) F6H0.6; (**E**,**e**) F6H0.8; (**F**,**f**) F6H1.0.

Impact strength is a critical indicator of the mechanical properties of polymer materials. To study the effect of the vibration force field on the impact strength of the PLA/PBS/EGMA blend, scanning electron microscopy (SEM) was used to observe the impact fracture surfaces of samples prepared under steady-state and dynamic conditions. The microstructure of the impact fracture surface under different amplitudes is shown in Figure 5. Figure 5A,a shows the brittle fracture surface under the application of the vibration force field, where the majority of the surface exhibits a smooth appearance, indicating brittle behavior, while some regions show minor plastic deformation. When the amplitude is H = 0.2 mm, the microstructure of the impact fracture surface is shown in Figure 5B,b. The plastic deformation region increases, suggesting an improvement in the toughness of the blend. As the amplitude continues to increase, the phase morphology of the impact surface changes significantly. The area of plastic deformation increases noticeably, and hole formation occurs. Particularly, when the amplitude reaches H = 1.0 mm, as shown in Figure 5F,f, a large number of fibers are pulled out at the fracture surface. The entire area exhibits extensive plastic deformation, and more holes appear. The presence of plastic deformation and holes can absorb a significant amount of impact energy, indicating that the introduction of amplitude enhances the mixing ability of PLA, PBS, and EGMA, promoting in situ reactions between the three phases and improving the compatibility of the PLA/PBS/EGMA blend.

**Figure 6:**
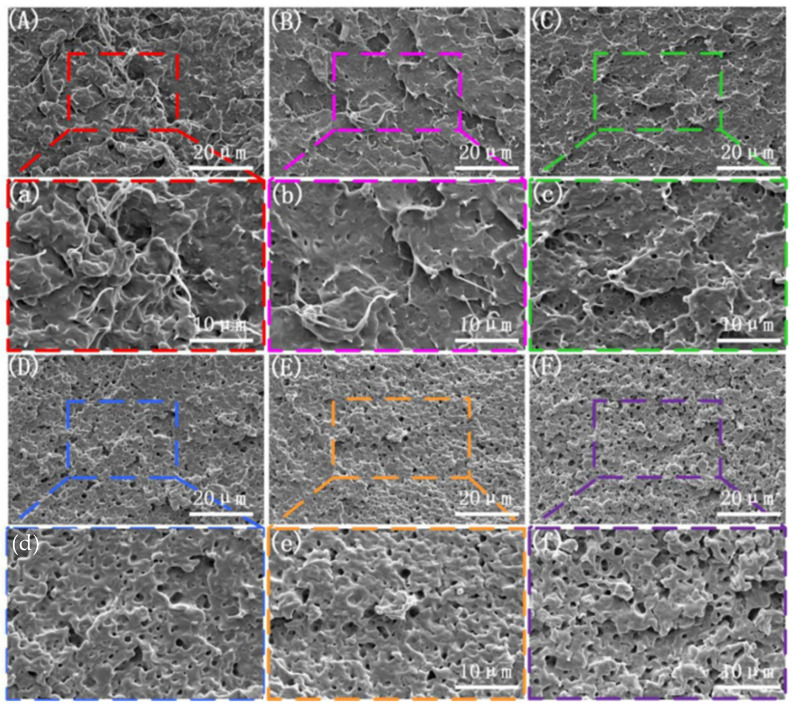
Microscopic morphology of impact fracture surfaces with different vibration frequencies: (**A**,**a**) F0H0; (**B**,**b**) H0.4F2; (**C**,**c**) H0.4F4; (**D**,**d**) H0.4F6; (**E**,**e**) H0.4F8; (**F**,**f**) H0.4F10.

The microstructure of the impact fracture surface under different vibration frequencies is shown in Figure 6. The pattern of change in the impact fracture surface with varying vibration frequency follows a similar trend to that observed with vibration amplitude. When the vibration force field is applied (with a fixed amplitude of H = 0.4 mm), at a frequency of F = 2 Hz, the impact surface shown in Figure 6B,b exhibits an increase in the plastic deformation region, with some holes appearing. When the vibration frequency is increased to F = 4 Hz, as shown in Figure 6C,c, torn fibers appear in some areas of the impact surface, indicating an improvement in the toughness of the blend. As the frequency increases from F = 6 Hz to F = 10 Hz, shown in Figure 6D–F, the entire region exhibits large-scale plastic deformation, and more holes appear. The presence of plastic deformation and holes allows for the absorption of a significant amount of impact energy. This suggests that an increase in vibration frequency enhances the mixing ability of PLA, PBS, and EGMA, promoting in situ reactions among the three phases and improving the compatibility of the PLA/PBS/EGMA blend.

#### 3.2.2. The Influence of Vibration Force Field on the Gel Fraction of the System

The epoxy groups of EGMA react with the hydroxyl groups of PLA and PBS, forming a PLA/PBS-g-EGMA ternary graft copolymer. The generation of a crosslinked structure in the graft copolymer affects the mechanical properties of the PLA/PBS/EGMA system. To investigate the influence of the vibration field on the crosslinking structure, PLA/PBS/EGMA blends prepared under different vibration field parameters were dissolved in chloroform, and the resulting gel formed by EGMA was collected through filtration. The impact of the vibration field on the gel was then studied.

**Figure 7:**
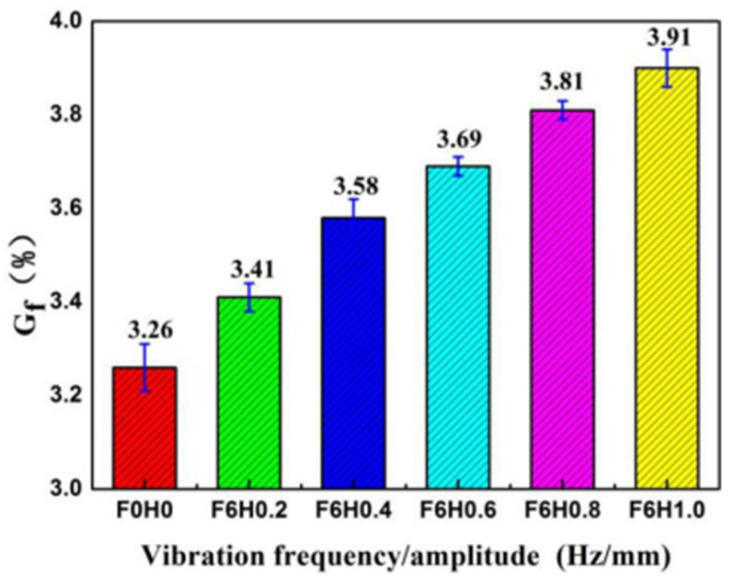
Gel fraction of PLA/PBS/EGMA blend under different vibration amplitudes.

The change in gel fraction of the PLA/PBS/EGMA blend under different vibration amplitudes is shown in Figure 7. Under steady-state working conditions, the gel fraction of the blend is 3.26%. When the vibration field is applied (with a fixed frequency of F = 6 Hz), the gel fraction increases with the amplitude, indicating that the reaction degree of the three-phase material increases. This leads to an increase in the amount of PLA/PBS-g-EGMA ternary graft copolymer. The main reason for this is that as the amplitude increases, the axial reciprocating motion of the middle screw increases, enhancing the compression and expansion of the three-phase material. The probability of contact between the three phases increases, allowing more of the material to react, which results in an increased gel fraction. Additionally, from the scanning electron microscope (SEM) images of the brittle fracture surface and etched surface, it can be observed that as the amplitude increases, the interface between the two phases reduces. This is primarily due to the increase in PLA/PBS-g-EGMA ternary graft copolymer, which is distributed at the interface of the PLA and PBS phases. This further confirms that the increase in the ternary graft copolymer leads to a higher gel fraction.

**Figure 8:**
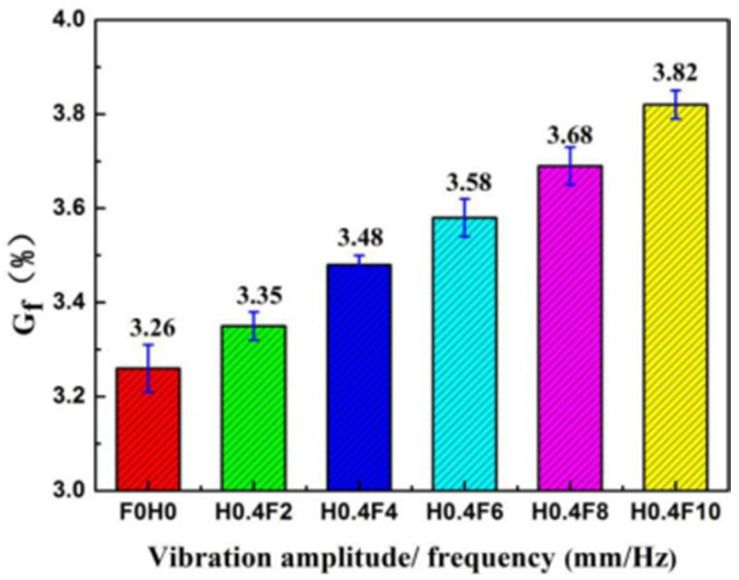
Gel fraction of PLA/PBS/EGMA blend under different vibration frequency.

The change in gel fraction of the PLA/PBS/EGMA blend under different vibration frequencies is shown in Figure 8. Similarly, the introduction of vibration frequency causes the gel fraction to increase as the frequency increases, indicating that the reaction degree of the three-phase material improves, leading to an increase in the amount of PLA/PBS-g-EGMA ternary graft copolymer. The main reason for this is that as the vibration frequency increases, the frequency of axial reciprocating motion of the middle screw per unit time also increases. The compression and expansion of the three-phase material also increase, which raises the probability of contact between the phases, thereby allowing more of the material to react and resulting in a higher gel fraction.

#### 3.2.3. The Influence of Vibration Force Field on the Mechanical Properties of the System

**Figure 9:**
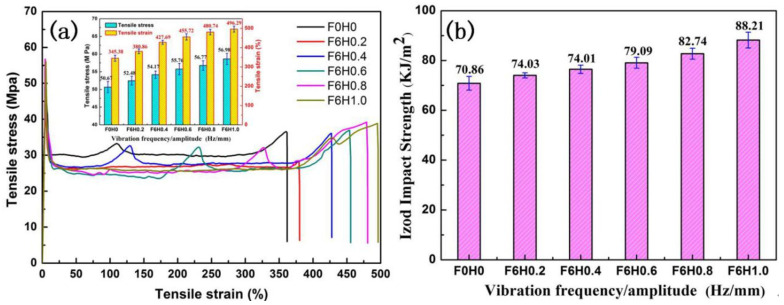
Effect of vibration amplitude on mechanical properties of PLA/PBS/EGMA blend: (**a**) strain–stress curves and tensile strength; (**b**) notched impact strength.

Mechanical properties are crucial indicators of polymer performance, significantly influencing the application of polymer materials. In Section 3.2.1 of this chapter, the impact of the vibration field on the microstructure of the prepared PLA/PBS/EGMA blend was studied, revealing the relationship between the material’s microstructure and its mechanical properties. This section focuses on analyzing the effect of different vibration amplitudes and frequencies on the tensile and impact properties of the PLA/PBS/EGMA blend, as shown in Figure 9.

The effect of different vibration amplitudes on the tensile properties of the PLA/PBS/EGMA blend is shown in Figure 9. Under steady-state conditions, the tensile strength and elongation at break of the blend are 50.67 MPa and 345.38%, respectively. When a vibration field is applied (with a fixed vibration frequency of F = 6 Hz), the tensile strength and elongation at break for different amplitudes (0.2 mm, 0.4 mm, 0.6 mm, 0.8 mm, 1.0 mm) are as follows:

- Tensile strength: 52.48 MPa, 54.17 MPa, 55.76 MPa, 56.77 MPa, 58.63 MPa.
- Elongation at break: 380.66%, 427.69%, 455.72%, 480.74%, 496.29%.

This indicates that both tensile strength and elongation at break increase with the increase in amplitude. The primary reason for this trend is that, without applying vibration, poor mixing results in uneven dispersion, leading to the aggregation of PBS and EGMA particles. This prevents the three polymers from fully interacting and undergoing in situ reactions. Because PLA and PBS are incompatible, visible gaps appear between the PLA matrix and PBS, and clear phase boundaries exist, causing stress concentration during stretching, which leads to breakage. As the amplitude increases, the powerful shear effect generated by the rotating screw in the extruder breaks and compresses the PBS and EGMA particles, enhancing their interaction through shear, stretching, and compression. The increase in amplitude improves the movement of polymer molecular chains, making the dispersed PBS and EGMA particles smaller and more uniform, and increasing the contact area between the three phases, promoting in situ reactions. This results in an increase in the tensile strength and elongation at break of the PLA/PBS/EGMA blend.

The effect of different vibration amplitudes on the impact strength of the PLA/PBS/EGMA blend is shown in Figure 9b. Under steady-state conditions, the impact strength of the blend is 70.86 KJ/m^2^. When a vibration field is applied (with a fixed vibration frequency of F = 6 Hz), the impact strength increases with the amplitude, especially when the amplitude reaches 1.0 mm, where the impact strength reaches 88.21 KJ/m^2^. This increase is attributed to the enhanced mixing effect caused by the vibration, which allows the three-phase system to fully react. Scanning electron microscopy images of the fracture surface show that EGMA effectively connects the incompatible PLA and PBS phases, improving the compatibility of the blend. As a result, when the sample is impacted, a large number of fibers and voids are observed. This plastic deformation can absorb significant amounts of impact energy, thereby increasing the impact strength.

**Figure 10:**
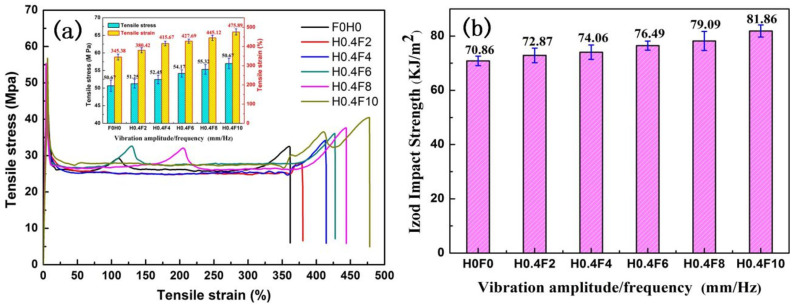
Effect of vibration frequency on mechanical properties of PLA/PBS/EGMA blend: (**a**) strain–stress curves and tensile strength (**b**) notched impact strength.

The effect of different vibration frequencies on the tensile strength and elongation at break of the PLA/PBS/EGMA blend is shown in Figure 10a. Under steady-state conditions, the tensile strength and elongation at break of the blend are 50.67 MPa and 345.38%, respectively. After the introduction of the vibration field, the tensile strength and elongation at break for vibration frequencies of 2 Hz, 4 Hz, 6 Hz, 8 Hz, and 10 Hz are as follows:

- Tensile strength: 51.25 MPa, 52.45 MPa, 54.17 MPa, 55.32 MPa, 56.67 MPa.
- Elongation at break: 380.42%, 415.67%, 427.69%, 445.12%, 475.89%.

These results show that the trend of increasing tensile strength and elongation at break with increasing vibration frequency is similar to that of increasing amplitude. The primary reason for this increase is that the vibration frequency increases the number of axial pulsating stretching–compression cycles per unit of time, improving the mixing frequency. This accelerates the movement of the polymer molecular chains, causing the dispersed PBS and EGMA particles to break into smaller, more uniform particles, increasing the contact area between the three-phase polymers. This allows for more in situ reactions, thus improving the tensile strength and elongation at break of the PLA/PBS/EGMA blend.

Figure 10b shows the effect of different vibration frequencies on the impact strength of the PLA/PBS/EGMA blend. Under steady-state conditions, the impact strength of the blend is 70.86 KJ/m^2^. After the introduction of the vibration field, the impact strength increases with the vibration frequency, particularly when the frequency reaches 10 Hz, where the impact strength reaches 81.86 KJ/m^2^. This increase is attributed to the improved mixing effect induced by the vibration frequency, which allows the three-phase system to fully react. Scanning electron microscopy images of the fracture surface reveal that, due to uniform mixing, EGMA effectively connects the incompatible PLA and PBS phases, improving the compatibility of the blend. As the vibration frequency increases, the improved compatibility results in the appearance of a large number of fibers and voids when the sample is impacted. This plastic deformation can absorb significant amounts of impact energy.

In conclusion, the introduction of a vibration field significantly enhances the mechanical properties of the PLA/PBS/EGMA blend. Combined with the previous section on the impact of the vibration field on the microstructure, the results show that the vibration field improves the mixing effect, providing a new approach for polymer processing and molding.

#### 3.2.4. The Influence of Vibration Force Field on the Crystallization Behavior of the System

**Figure 11:**
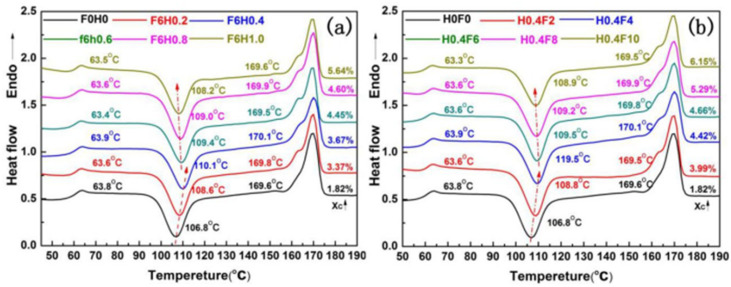
DSC curve of PLA/PBS/EGMA samples with vibrational force field: (**a**) vibration amplitude (**b**) vibration frequency.

**Figure 12:**
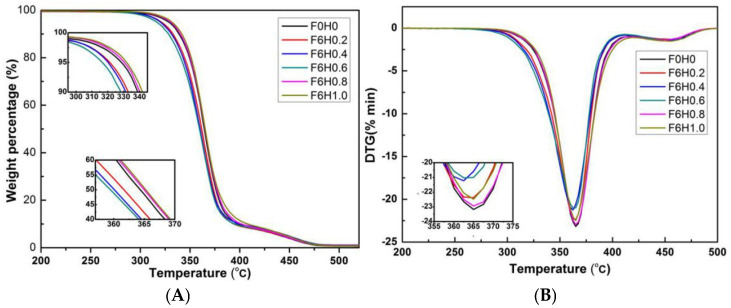
Thermal-stabilities of PLA/PBS/EGMA ternary blends with different vibration amplitudes: (**A**) TGA curves; (**B**) DTG curves.

The analysis of crystallization behavior provides a good measure of the crystallization ability of polymer materials and reflects the molecular chain mobility of the material. The second heating scan of the differential scanning calorimetry (DSC) curve under different vibration frequencies and amplitude parameters is shown in Figure 11 and Figure 12. From the DSC curves, the glass transition temperature (Tg), cold crystallization temperature (Tcc), melting temperature (Tm), and crystallinity (*λc*) can be obtained.

Figure 11a shows the DSC curves of the PLA/PBS/EGMA blend under different amplitudes. Under steady-state conditions, the glass transition temperature (Tg) of the PLA/PBS/EGMA blend is 63.8 °C, which corresponds to the Tg of PLA. After the introduction of vibration, the glass transition temperatures of the blend at amplitudes of 0.2 mm, 0.4 mm, 0.6 mm, 0.8 mm, and 1.0 mm are 63.6 °C, 63.6 °C, 63.9 °C, 63.4 °C, and 63.5 °C, respectively. With increasing amplitude, there is a slight downward trend in Tg. Since EGMA has a lower Tg, the glass transition temperature shifts to lower temperatures, indicating that the increase in amplitude improves the compatibility of the blend.

The cold crystallization temperature (Tcc) of the PLA/PBS/EGMA blend without vibration is 106.8 °C. With amplitudes of 0.2 mm, 0.4 mm, 0.6 mm, 0.8 mm, and 1.0 mm, the cold crystallization temperatures are 108.6 °C, 110.1 °C, 109.4 °C, 109.0 °C, and 108.2 °C, respectively. The results indicate that the introduction of vibration causes a significant shift of the cold crystallization temperature to higher temperatures, suggesting that the addition of amplitude limits the movement of the PLA molecular chains. This is mainly due to the improved dispersion effect of the PLA/PBS/EGMA blend caused by the vibration, promoting a more uniform dispersion of the three-phase system. As a result, EGMA can more effectively react in situ with PLA and PBS at the interface, enhancing the compatibility while simultaneously restricting the movement of the molecular chains. Moreover, the increase in Tcc is most noticeable when the amplitude reaches 0.4 mm, and when the amplitude exceeds 0.4 mm, there is a slight decrease in Tcc. This could be attributed to the relatively high content of 8% EGMA in the system, as not all of it participates in the in situ reaction at the interface. Despite the slight decrease in Tcc, it still remains higher than that of the blend processed under steady-state conditions.

The melting temperature (Tm) follows a trend of initially increasing and then decreasing, which is similar to the changes observed in the glass transition temperature. The reasons for this are analogous and will not be discussed here.

As for the crystallinity (Xc) of the PLA/PBS/EGMA blend, the crystallinity of the blend without vibration is 1.82%. With the increase in amplitude, the crystallinity increases. This is mainly due to the fact that the addition of vibration causes the blend to experience some orientation, and as the amplitude increases, the orientation becomes more pronounced. This enhances the molecular chain order, accelerates the formation and growth of PLA crystallites, and results in an increase in crystallinity.

Figure 11b presents the DSC curves of the PLA/PBS/EGMA blend under different vibration frequencies. Under steady-state conditions, the glass transition temperature (Tg) of the PLA/PBS/EGMA blend is 63.8 °C, corresponding to the Tg of PLA. When a vibration field is applied (with a fixed amplitude of H = 0.4 mm), the Tgs of the blend at vibration frequencies of 2 Hz, 4 Hz, 6 Hz, 8 Hz, and 10 Hz are 63.6 °C, 63.9 °C, 63.6 °C, 63.6 °C, and 63.3 °C, respectively. There is a slight downward trend in Tg as the vibration frequency increases. Since EGMA has a lower Tg, the shift in the glass transition temperature to lower temperatures indicates that the increase in vibration frequency improves the blend’s compatibility. Under steady-state conditions, the cold crystallization temperature (Tcc) of the PLA/PBS/EGMA blend is 106.8 °C. With vibration frequencies of 2 Hz, 4 Hz, 6 Hz, 8 Hz, and 10 Hz, the cold crystallization temperatures are 108.8 °C, 109.5 °C, 109.5 °C, 109.2 °C, and 108.9 °C, respectively. The results show that the introduction of vibration frequency causes the cold crystallization temperature (Tcc) of the blend to shift significantly to higher temperatures. This suggests that the addition of vibration frequency hinders the movement of PLA molecular chains. The primary reason for this is that the introduction of vibration frequency improves the dispersion effect of the PLA/PBS/EGMA blend, promoting the uniform dispersion of the three-phase system. EGMA can then fully interact with PLA and PBS, facilitating in situ interfacial reactions and enhancing compatibility, which, in turn, restricts the mobility of molecular chains. Additionally, the figure shows that when the vibration frequency increases to 6 Hz, the increase in Tcc is very noticeable. However, when the vibration frequency exceeds 6 Hz, there is a slight decrease in Tcc. This could be due to the relatively high content of 8% EGMA, where not all of it participates in the in situ interfacial reaction. As the vibration frequency increases beyond a certain point, the mixture becomes more homogeneous. Since EGMA is an elastomer, further increasing the vibration frequency may cause EGMA to act as a plasticizer, enhancing the molecular chain’s mobility. Although Tcc decreases slightly when the vibration frequency exceeds 6 Hz, it remains higher than the temperature under steady-state conditions. The melting temperature (Tm) also shows an initial increase followed by a decrease, with reasons similar to the changes in the glass transition temperature, and will not be repeated here. As for the crystallinity (Xc) of the PLA/PBS/EGMA blend, the crystallinity of the blend without vibration is 1.82%. With the increase in vibration frequency, the crystallinity increases. This is mainly because the introduction of vibration frequency leads to some orientation in the blend, which becomes more pronounced as the vibration frequency increases. This orientation enhances the molecular chain order, accelerates the formation and growth of PLA crystallites, and results in an increase in crystallinity.

#### 3.2.5. The Influence of Vibration Force Field on the Thermal Stability of the System

This section analyzes the impact of the vibration field on the thermal stability of the PLA/PBS/EGMA ternary blend. The temperatures at which 5% (T5) and 50% (T50) weight loss occurs represent the initial degradation temperature and the intermediate degradation temperature, respectively, which can be obtained from the TGA (Thermogravimetric Analysis) curve. The temperature corresponding to the maximum degradation rate (Tmax) can be measured from the DTG (Derivative Thermogravimetric Analysis) curve. By analyzing the TGA and DTG curves, the thermal stability of the material can be assessed under different conditions, including the application of vibration fields. The T5, T50, and Tmax provide key information about the thermal degradation process of the PLA/PBS/EGMA blend, helping to understand the influence of vibration fields on the polymer’s thermal behavior and stability.

Figure 12 presents the TGA and DTG curves for the thermal stability of the PLA/PBS/EGMA ternary blend under varying amplitudes. As shown in Figure 12 and Table 4, when no vibration amplitude is applied, the T5, T50, and Tmax of the ternary blend are 328.6 °C, 364.6 °C, and 365.3 °C, respectively. After the introduction of vibration amplitude, these three degradation temperatures show a decreasing trend. When the amplitude is below 0.6 mm, this indicates that the amplitude lowers the thermal stability of the blend. However, as the amplitude further increases above 0.6 mm, the T5, T50, and Tmax begin to rise. Specifically, at an amplitude of 1.0 mm, these degradation temperatures are 333.7 °C, 365.2 °C, and 365.1 °C, showing that higher amplitudes can enhance the thermal stability of the PLA/PBS/EGMA ternary blend. This suggests that a higher vibration amplitude contributes to the improvement in the overall thermal stability of the material, potentially due to better mixing and more effective interaction between the components.

**Figure 13:**
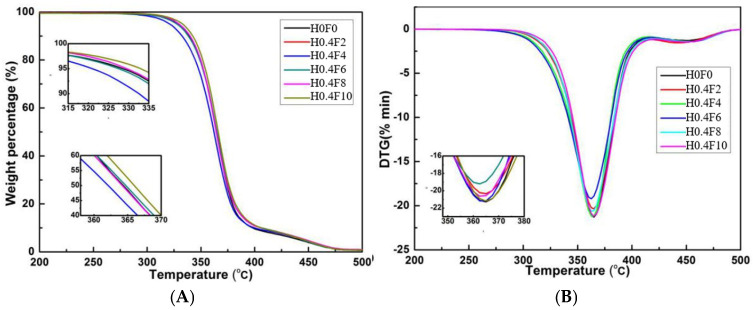
Thermal-stabilities of PLA/PBS/EGMA ternary blends with different vibration frequency: (**A**) TGA curves; (**B**) DTG curves.

Figure 13 presents the TGA and DTG curves for the thermal stability of the PLA/PBS/EGMA ternary blend under varying vibration frequencies. As shown in Figure 13 and Table 5, when no vibration frequency is applied, the T5, T50, and Tmax of the ternary blend are 328.6 °C, 364.6 °C, and 365.3 °C, respectively. After the introduction of vibration frequency, these three degradation temperatures show a decreasing trend. When the vibration frequency is below 6 Hz, this indicates that the vibration frequency reduces the thermal stability of the blend. However, as the vibration frequency increases above 6 Hz, the T5, T50, and Tmax start to rise. Specifically, at a vibration frequency of 10 Hz, these degradation temperatures are 333.1 °C, 366.0 °C, and 365.9 °C, showing that higher vibration frequencies can enhance the thermal stability of the PLA/PBS/EGMA ternary blend. This suggests that higher vibration frequencies contribute to improved thermal stability, likely due to better mixing and a more uniform distribution of components in the blend.

**Figure 14:**
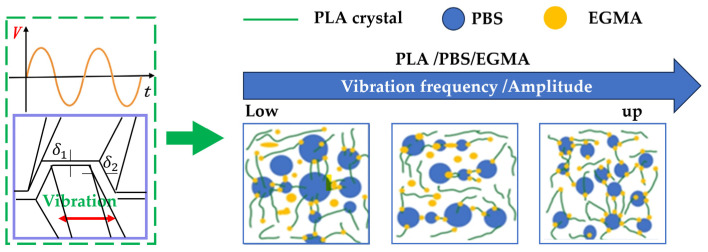
Dispersion and mixing mechanism of PLA/PBS/EGMA blends under vibration force field.

The dispersion and mixing mechanism of the PLA/PBS/EGMA blend under the influence of vibration fields is shown in Figure 14. From both theoretical analysis and experimental results in this chapter, it is observed that the introduction of the vibration field generates a shear-stretch composite flow field, which improves the dispersion and mixing effect. As the vibration amplitude and frequency increase, the particle size of PBS and EGMA decreases. At the same time, the proportion of in situ reactions between the epoxy groups of EGMA and the hydroxyl groups in the PLA and PBS molecules increases, leading to a higher amount of PLA/PBS-g-EGMA copolymer distributed at the interface between PLA and PBS phases. This significantly improves the compatibility of the three-phase materials, thereby enhancing the gel fraction, mechanical properties, and thermal stability of the PLA/PBS/EGMA blend.

#### 3.2.6. The Dispersion and Mixing Mechanism of the Action of the Vibration Force Field on the System

The dispersion and mixing mechanism of the vibration force field’s action on the PLA/PBS/EGMA blend is illustrated in Figure 14. Figure 14 was drawn based on the results of scanning electron microscopy. Through theoretical analysis and experimental analysis in this paper, it is found that when the vibration force field is introduced into the PLA/PBS/EGMA blend system, a shear-stretching composite flow field is generated. This composite flow field can exert complex forces on the particles within the blend, making it easier to disperse the originally agglomerated particles. As shown in the figure, as the amplitude and vibration frequency increase from low to high, the particle sizes of PBS and EGMA gradually decrease, indicating a remarkable dispersion effect of the composite flow field. The reduction in particle size helps to enhance the uniformity of the blend, thereby improving the overall properties of the material. Under the action of the vibration force field, the proportion of in situ reactions between the epoxy groups of EGMA and the hydroxyl groups in PLA molecules and PBS molecules within the blend increases. This is because the vibration force field increases the collision probability between molecules, facilitating the occurrence of the reaction and leading to the generation of more PLA/PBS-g-EGMA copolymers. These copolymers are distributed at the phase interface between PLA and PBS. The increase in copolymers at the phase interface enhances the interaction between the two phases and improves the compatibility of the three-phase material. Good compatibility has a positive impact on the gel fraction, mechanical properties, and thermal stability of the blend.

## 4. Conclusions

Based on the experimental results under steady-state force fields, the 82/10/8 composition was selected as the research subject. Based on the experimental results under the action of a steady-state force field, the 82/10/8 composition was selected as the research object. Through the orthogonal experimental design and entropy method analysis, the influences of vibration field parameters and process parameters on the comprehensive mechanical properties of the blend were investigated. The results show that the vibration amplitude and frequency have a significant impact on the overall mechanical properties, while the processing temperature and rotational speed have little influence. SEM observation reveals that the vibration force field reduces the size of PBS particles and makes their distribution more uniform. The “sea-island” structure gradually disappears, and the phase interface is fused, which enhances the compatibility. When the amplitude and frequency increase, the plastic deformation region of the blend expands, and more fibers and holes appear, enhancing the ability to absorb impact energy. As the vibration amplitude and frequency increase, the tensile strength, elongation at break, and impact strength of the blend all increase. When the amplitude is 1.0 mm, the impact strength increases from 70.86 KJ/m^2^ in the steady state to 88.21 KJ/m^2^; when the frequency is 10 Hz, the impact strength reaches 81.86 KJ/m^2^. DSC analysis indicates that vibration slightly decreases the glass transition temperature (Tg), increases the cold crystallization temperature (Tcc), and raises the crystallinity (Xc). For example, when the amplitude increases, Xc gradually increases from 1.82% without vibration. The TGA and DTG curves show that when the vibration amplitude and frequency are lower than a certain value, the thermal stability of the blend decreases; when they are higher than that value, the thermal stability is enhanced. For instance, when the amplitude is 1.0 mm, the initial degradation temperature (T5) increases from 328.6 °C to 333.7 °C; when the frequency is 10 Hz, T5 rises to 333.1 °C. The experimental results show that the introduction of the vibration field generates a shear-stretching composite flow field, enhancing the dispersion effect. This improves the in situ reaction between the epoxy groups of EGMA and the hydroxyl groups in PLA and PBS molecules, and increases the distribution amount of the PLA/PBS-g-EGMA copolymer at the phase interface between PLA and PBS. This indicates that the compatibility of the three-phase material is significantly improved, resulting in an increase in the gel fraction, mechanical properties, and thermal stability of the PLA/PBS/EGMA blend.

Considering the enhanced properties of the PLA/PBS/EGMA blend, it holds potential application prospects in various fields. In the packaging industry, due to its improved mechanical properties and thermal stability, it can be used to manufacture high-performance packaging materials for products that require better protection during storage and transportation.

## Figures and Tables

**Table 1:** Orthogonal experiment factors and levels.

Factors	Temperature	Rotational Speed	Vibration Frequency	Amplitude
Level	(A)	(B)	(C)	(D)
1	190 °C	70 r/min	2 Hz	0.2 mm
2	200 °C	80 r/min	4 Hz	0.4 mm
3	210 °C	90 r/min	6 Hz	0.6 mm
4	220 °C	100 r/min	8 Hz	0.8 mm

**Table 2:** As the index of displacement analysis form intuitive.

Experimental Number and Factors	A	B	C	D	Empty	Tensile Strength (MPa)	Elongation at Break (%)	Impact Strength (KJ/m^2^)	Comprehensive Score
1	1	1	1	1	1	42.01	458.06	62.94	88.51
2	1	2	2	2	2	41.28	475.41	66.31	92.44
3	1	3	3	3	3	41.3	432.42	63.36	86.99
4	1	4	4	4	4	41.2	432.73	60.44	84.58
5	2	1	2	3	4	44.03	465.23	52.57	80.60
6	2	2	1	4	3	36.94	458.07	68.18	92.35
7	2	3	4	1	2	41.67	448.39	60.81	86.03
8	2	4	3	2	1	41.31	444.07	63.14	87.62
9	3	1	3	4	2	41.77	468.2	53.86	81.65
10	3	2	4	3	1	40.41	462.9	63.33	89.01
11	3	3	1	2	4	42.09	479.95	70.46	96.29
12	3	4	2	1	3	42.62	462.74	68.02	93.11
13	4	1	4	2	3	42.69	464.53	51.79	79.77
14	4	2	3	1	4	41.81	462.91	67.94	92.97
15	4	3	2	4	1	42.49	470.51	58.12	85.42
16	4	4	1	3	2	41.79	469.88	74.79	99.15
K1j	88.130	89.030	94.075	86.633	87.640				
K2j	86.650	90.155	87.892	91.692	89.817				
K3j	90.015	88.938	87.308	86.683	88.055				
K4j	89.328	86.000	84.847	91.115	86.610				
Range	3.35	4.155	9.228	9.059	2.177				
set priorities	C > D > B > A								

**Table 3:** ANOVA table.

Factors	Sum of Squares of Deviations	Degree of Freedom f	Mean Square Sum S	The Value of F	Significant Value
A	26.143	3	2.437	9.280	
B	37.830	3	3.527	9.280	
C	184.835	3	17.231	9.280	*
D	205.950	3	19.199	9.280	*
error	10.73	3			

For a given significance level α = 0.05, * means that this factor has a significant influence on the experimental results.

**Table 4:** Thermal stability parameters with different vibration amplitudes.

Sample	T_5%_ (°C)	T_50%_ (°C)	T_max_ (°C)
F0H0	328.6	364.6	365.3
F6H0.2	321.2	361.7	363.6
F6H0.4	320.1	360.1	362.3
F6H0.6	316.5	359.5	363.7
F6H0.8	330.5	365.0	365.2
F6H1.0	331.7	365.2	365.1

**Table 5:** Thermal stability parameters with different vibration frequencies.

Sample	T_5_% (°C)	T_50_% (°C)	T_max_ (°C)
H0f0	328.6	364.6	365.2
H0.4f2	327.6	364.7	364.2
H0.4f4	321.0	362.1	363.6
H0.4f6	320.0	361.7	362.3
H0.4f8	329.8	364.2	363.6
H0.4f10	333.1	366.0	365.9

## Data Availability

Data are contained within the article.
